# Effects of early adversity and social discrimination on empathy for complex mental states: An fMRI investigation

**DOI:** 10.1038/s41598-019-49298-4

**Published:** 2019-09-10

**Authors:** Melike M. Fourie, Dan J. Stein, Mark Solms, Pumla Gobodo-Madikizela, Jean Decety

**Affiliations:** 1 Studies in Historical Trauma and Transformation, Stellenbosch University, Stellenbosch, South Africa; 2 Department of Psychiatry and MRC Unit on Risk & Resilience in Mental Disorders, University of Cape Town, Cape Town, South Africa; 3 Department of Psychology, University of Cape Town, Cape Town, South Africa; 4 Department of Psychology and Department of Psychiatry and Behavioral Neuroscience, University of Chicago, Chicago, IL, United States

**Keywords:** Amygdala, Empathy, Stress and resilience

## Abstract

There is extensive evidence of an association between early adversity and enduring neural changes that impact socioemotional processing throughout life. Yet little is known about the effects of on-going social discrimination on socioemotional functioning. Here we examined how cumulative experiences of social discrimination impact brain response during empathic responding—a crucial issue in South Africa, given its historical apartheid context and continuing legacies. White and Black South Africans completed measures of social adversity (early adversity and social discrimination), and underwent fMRI while viewing video clips depicting victims and perpetrators of apartheid crimes. Increased neural response was detected in brain regions associated with cognitive rather than affective empathy, and greater social adversity was associated with reduced reported compassion across participants. Notably, social discrimination (due to income level, weight, gender) in White participants was associated with increased amygdala reactivity, whereas social discrimination (due to race) in Black participants mediated the negative associations of temporoparietal junction and inferior frontal gyrus activation with compassion during emotionally provocative conditions. These findings suggest that (i) social discrimination has comparable associations at the neural level as other psychosocial stressors, and that (ii) the mechanisms underlying empathic responding vary as a function of the type of social discrimination experienced.

## Introduction

Early social experiences critically shape the neural circuitry underpinning social and emotional behavior throughout life^1^. For example, converging evidence suggests that early adversity, such as physical or emotional abuse, is associated with enduring changes in brain structure and function in circuits that facilitate stress responsivity, including the amygdala and anterior cingulate cortex (ACC), giving rise to alterations in emotional processing and regulation^2–4^. In contrast to this rich literature, our understanding of the impact of experiences of racism and social discrimination on brain functioning in marginalized groups remains poor, despite growing evidence of the immense toll of chronic experiences of such discrimination (actual and perceived) on physical and mental well-being^5–8^.

Perceived discrimination is an enduring form of psychosocial stress with cumulative effects on health^9,10^. Although overt racial discrimination may be declining worldwide, racial disparities are maintained by subtler forms of discrimination (e.g., ingroup favouritism, microaggression), which appears to be equally consequential in terms of psychological consequences^11–13^. Moreover, research is only beginning to unravel the neural mechanisms underlying the intergenerational transmission of systematic and long-standing forms of discrimination and collective violence^14,15^.

These are important issues for South Africa—more than two decades after its first democratic elections, the wounds and historical divisions resulting from gross human rights violations during apartheid remain deeply entrenched there. While much research has focused on intergroup responding in post-conflict countries, here we wanted to explore how cumulative experiences of social adversity impact neural circuitry underlying one of the most critical of human sensitivities, namely the ability to empathize. Notably, empathy can facilitate psychological repair when people bear witness to trauma testimonies, including expressions of forgiveness and remorse^16^. If the mechanisms underlying empathy are disrupted, this could be costly not only at the societal level of relating to and caring for others^17^, but also at the individual level of regulating personal distress^18^.

Previous behavioral work suggests that psychosocial stress, including early and collective experiences of adversity, are associated with impaired empathy^19–22^. To extend such findings, we used fMRI to examine empathy for complex mental states in a sample of Black and White South Africans with no history of mental illness who experienced apartheid. Specifically, we examined empathy in response to ecological video clips depicting victims (forgiving/ unforgiving) and perpetrators (apologetic/unapologetic) of apartheid crimes, and determined the extent to which experiences of early adversity and social discrimination are associated with altered responses in neural circuits involved in cognitive and affective empathy. We speculated that these different social stressors may converge via shared psychological mechanisms impacting overlapping neural circuits, particularly in marginalized populations living in environments with complex social challenges, such as poverty, racial stratification, negative social connectedness, and early exposure to these conditions^23^.

Theoretical conceptualisations of empathy emphasize two complementary, yet dissociable, pathways to share and understand others’ emotional states: (1) a mechanism for affect sharing including the dorsal anterior cingulate cortex (dACC), anterior insula (aINS), amygdala and brainstem, and (2) a more reflective and cognitively effortful process using mentalizing capacities^24–28^. Whereas observing others in physical pain reliably activates the affective empathy network^29,30^, observing others in emotional distress more consistently recruits areas associated with mentalizing, including dorsal and ventral medial prefrontal cortex (dmPFC and vmPFC), temporoparietal junction (TPJ), posterior superior temporal sulcus (pSTS), precuneus, and posterior cingulate cortex (PCC)^31–34^. While the contribution of affective and cognitive processes likely varies depending on the empathy-eliciting situation^35^, it is plausible that the more effortful cognitive mechanism, which also taps into memory and other self-reflective processes, is readily engaged in response to complex mental states^31,36^.

Early adversity has been found to be associated with more reactive, and less sensitive empathic engagement^37,38^. At the anatomical level, early adversity has consistently been associated with gray matter volume changes in the hippocampus and amygdala (areas with high densities of glucocorticoid receptors involved in the stress response), as well as with frontolimbic circuitry irregularities, including heightened activation of the ACC, orbitofrontal cortex, and amygdala (particularly on the right)^2,39–42^. Early adversity affects a broader network of structures, which have received comparatively less attention. Notably, recent research documents increased gray matter volume or heightened responsivity to socioaffective cues in areas associated with social information processing, such as the posterior STS/TPJ, temporal pole, inferior frontal gyrus (IFG), and precuneus following early adversity^4,43–45^. It has been argued that such higher-order association cortices may be particularly vulnerable to the long-term effects of early adversity due to their protracted postnatal development^3^.

With regard to experiences of social discrimination, only a handful of studies have investigated the downstream biological structural and functional changes that may contribute to race-based health disparities^46–48^. Initial evidence from these studies parallels that of other psychosocial stressors, suggesting that social discrimination is associated with functional alterations in activation and connectivity of the amygdala and ACC, in particular^49^. These areas form part of the paralimbic “salience network” (SN), which coactivates in response to varied forms of personal salience^50,51^. On this account, the cumulative effects of discriminatory events might result in heightened sensitivity to racial issues, altered salience attribution, and hence increased susceptibility to everyday stressors. Nevertheless, no study has investigated how chronic experiences of different types of discrimination impact real-life empathic responding.

## Study Aims

First, the present study examined the neural response associated with empathy for complex mental states using ecological stimuli in adult Black and White South Africans. We hypothesized that neural activation in response to forgiving/unforgiving victims and apologetic/unapologetic perpetrators would be characterized by more effortful cognitive perspective taking, and thus activation in mentalizing areas (dmPFC, TPJ, precuneus), rather than by affective sharing mechanisms. Furthermore, we anticipated significantly higher reported compassion (empathic concern) for victims than for perpetrators^52^, particularly unapologetic perpetrators.

Second, we examined whether neural processing of material reminiscent of apartheid atrocities would be processed differently in Black compared to White participants, given that Black individuals endured historical oppression and marginalization. It was predicted that Black participants would respond with greater empathic arousal (sensitivity), as the evocative video clips might be of heightened personal relevance and emotional salience to them. Hence, we expected elevated activation in the affective empathy or “salience network”^49^, including the dACC, aINS, and other subcortical and limbic structures^53^.

Finally, we examined associations between specific forms of social adversity (early adversity and social discrimination) and reported compassion, as well as functional changes in cognitive and affective empathy mechanisms. In light of previous findings, we expected social adversity to be associated with reduced reported compassion^19^. At the neural level, we hypothesized that it would be associated with heightened activation in stress-related circuitry (amygdala) and areas involved in social information processing (temporal regions and IFG), thus affecting key areas associated with both cognitive and affective empathy. Because of the relative dearth of studies investigating neural alterations associated with social discrimination, our hypotheses are exploratory in nature.

## Results

### Behavioral data

Statistical analyses were carried out using IBM SPSS Statistics for Windows, version 25.0 (IBM Corp., Armonk, N.Y., USA).

#### Emotion ratings

**Figure 1:**
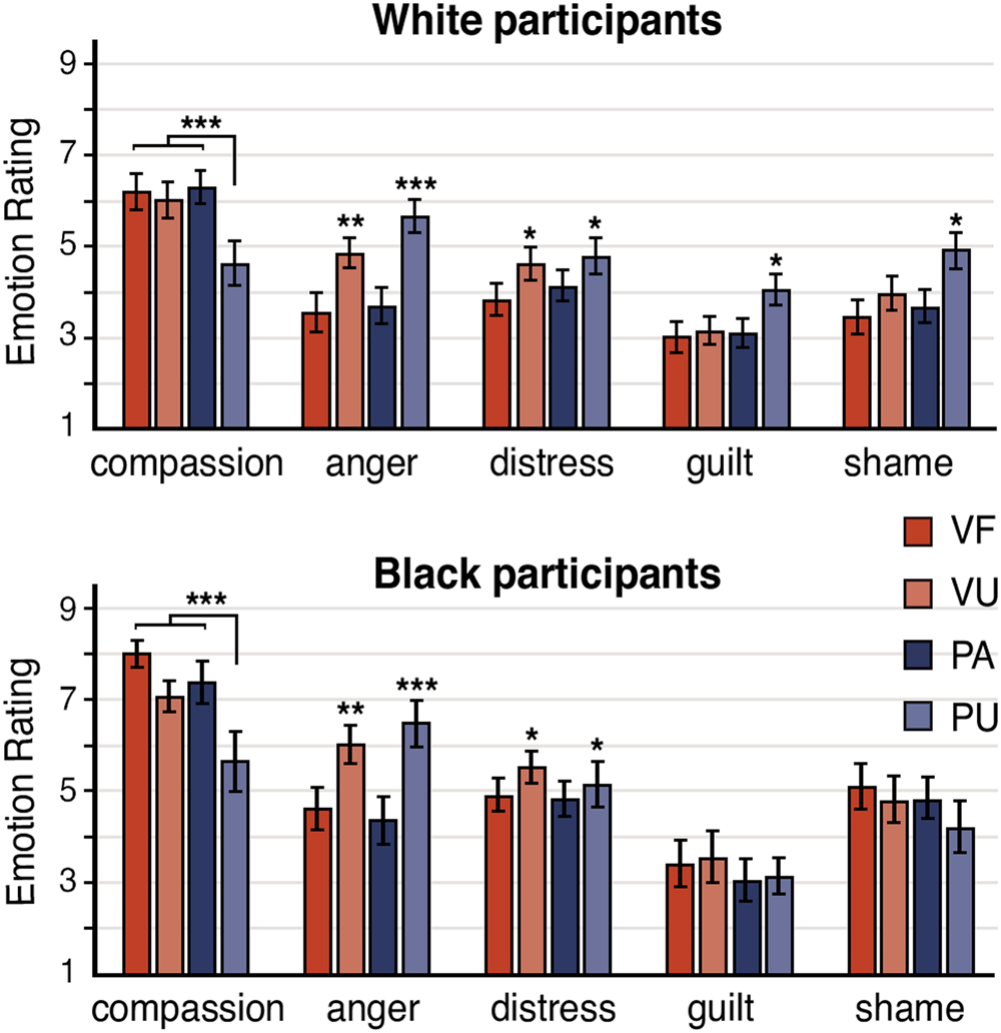
Subjective emotion ratings for participants in response to the 4 conditions of interest: victim forgiving (VF), victim unforgiving (VU), perpetrator apologetic (PA) and perpetrator unapologetic (PU). Note that anger represents moral indignation. Error bars indicate standard error of the mean. *p < 0.05. **p < 0.01. ***p < 0.001.

Post-scan emotion ratings were subjected to repeated-measures factorial ANOVAs (Fig. 1, Supplementary Table S3). In instances where the assumption of sphericity was violated, the degrees of freedom were adjusted using Greenhouse-Geisser epsilon corrections. We first examined the four conditions of interest, namely victim forgiving (VF), victim unforgiving (VU), perpetrator apologetic (PA), and perpetrator unapologetic (PU), using a 4 (Condition: VF, VU, PA, PU) × 5 (Emotion: compassion, moral indignation, personal distress, guilt, shame) repeated-measures factorial ANOVA. The main effect of condition was not significant (*p* = 0.11), but that of emotion was, *F*(2.92, 102.34) = 41.35, *p* < 0.001, *ε* = 0.73. Planned contrasts indicated that compassion was rated as significantly higher than all other emotions across conditions (*p*s < 0.001, *r*s > 0.73). The 2-way interaction was also significant, *F*(5.36, 187.68) = 13.00, *p* < 0.001, *ε* = 0.45.

Compassion was explored using a participant group × condition mixed factorial ANOVA. The main effect of participant group was significant (*F*(1, 33) = 7.05, *p* = 0.01), with Black participants’ ratings higher than those of White participants. The main effect of condition was also significant, *F*(2.06, 67.99) = 14.15, *p* < 0.001. As anticipated, contrasts indicated that compassion for the PU condition was significantly lower than all other conditions across participants (*p*s < 0.001, *r*s > 0.55), while compassion for the VF, VU, and PA conditions did not differ (*p*s > 0.16). A similar analysis for moral indignation indicated that the main effect of participant group was significant (*F*(1, 33) = 4.94, *p* = 0.03), with Black participants’ ratings higher than those of White participants. The main effect of condition was also significant, *F*(2.18, 72.07) = 15.26, *p* < 0.001. Contrasts indicated that ratings for the PU condition were significantly higher than those of the VF, PA (*p*s < 0.001, *r*s > 0.61) and VU (*p* = 0.03, *r* = 0.36) conditions, while the VU condition was rated higher in moral indignation than the VF and PA conditions (*p*s = 0.001, *r*s > 0.52). For personal distress, the main effect of condition was significant (*p* < 0.01), with contrasts indicating that PU and VU conditions were rated as higher than other conditions (*p*s < 0.05, *r*s > 0.35). Finally, for both guilt and shame, only the interactions between participant group and condition were significant (*F*s > 4.40, *p*s < 0.01), with contrasts revealing that White participants’ ratings were significantly higher than those of Black participants for PU compared to other conditions (*p*s < 0.05, *r*s > 0.32).

Post-hoc analysis confirmed that emotion ratings did not differ significantly as a function of victim or perpetrator race for either Black or White participants (see Supplementary Results).

#### Questionnaire measures

We detected significant negative correlations between participants’ reported social discrimination and compassion ratings in response to the VF, VU, and PA conditions (*r*_*35(2-tailed*)_ > −0.43, *p*s < 0.01) (see Supplementary Table S4).

While everyday discrimination scores were higher for Black than White participants, this difference was not significant (*p* = 0.13). There were significant qualitative differences in the perceived reasons for discrimination, however. Black participants reported race as the main reason (72%), whereas White participants reported gender (22%), income level (22%), and weight/appearance (22%), rather than race (17%), as the main reasons (Table 1).

**Table 1:** Social Adversity Reported by Participants.

Emotion	Black Participants (*n* = 18)	White Participants (*n* = 18)	Comparison of Means (*p*)
**Everyday Discrimination Scale**			
Total scores	23.11 (11 to 37)	19.39 (9 to 29)	0.13
% due to Race	72.22%	16.67%	**—**
% due to Gender	22.22%	22.22%	**—**
% due to Income level	27.80%	22.22%	**—**
% due to Age	16.67%	16.67%	**—**
% due to Weight/Appearance	16.67%	22.22%	**—**
% due to Sexual orientation	11.11%	11.11%	**—**
**Childhood Trauma Questionnaire**			
Total scores	40.89 (25 to 74)	41.33 (25 to 74)	0.92
Emotional abuse	8.06 (5 to 18)	8.89 (5 to 16)	0.37
Physical abuse	7.94 (5 to 18)	7.56 (5 to 15)	0.91
Sexual abuse	6.50 (5 to 13)	8.11 (5 to 25)	0.52
Emotional neglect	9.56 (5 to 18)	10.06 (5 to 15)	0.69
Physical neglect	8.83 (5 to 16)	6.72 (5 to 11)	0.17

*Note*. Data presented are means, with ranges in parentheses. *T*-tests were used to compare means. Where assumptions of normality were not met (as determined by Kolmogorov-Smirnov tests), non-parametric Mann-Whitney U tests were performed.

We also found significant negative correlations between participants’ childhood trauma (total and emotional abuse) scores and their compassion ratings in response to the VF and PA conditions (*r*_*35(2-tailed*)_ > −0.43, *p*s < 0.01) (see Supplementary Table S4). No significant differences for early adversity were reported by White and Black participants (*p*s > 0.16) (Table 1).

In general, greater social discrimination and early adversity, particularly emotional abuse, were associated with reduced reported compassion.

### fMRI data

#### Whole-brain contrasts

**Figure 2:**
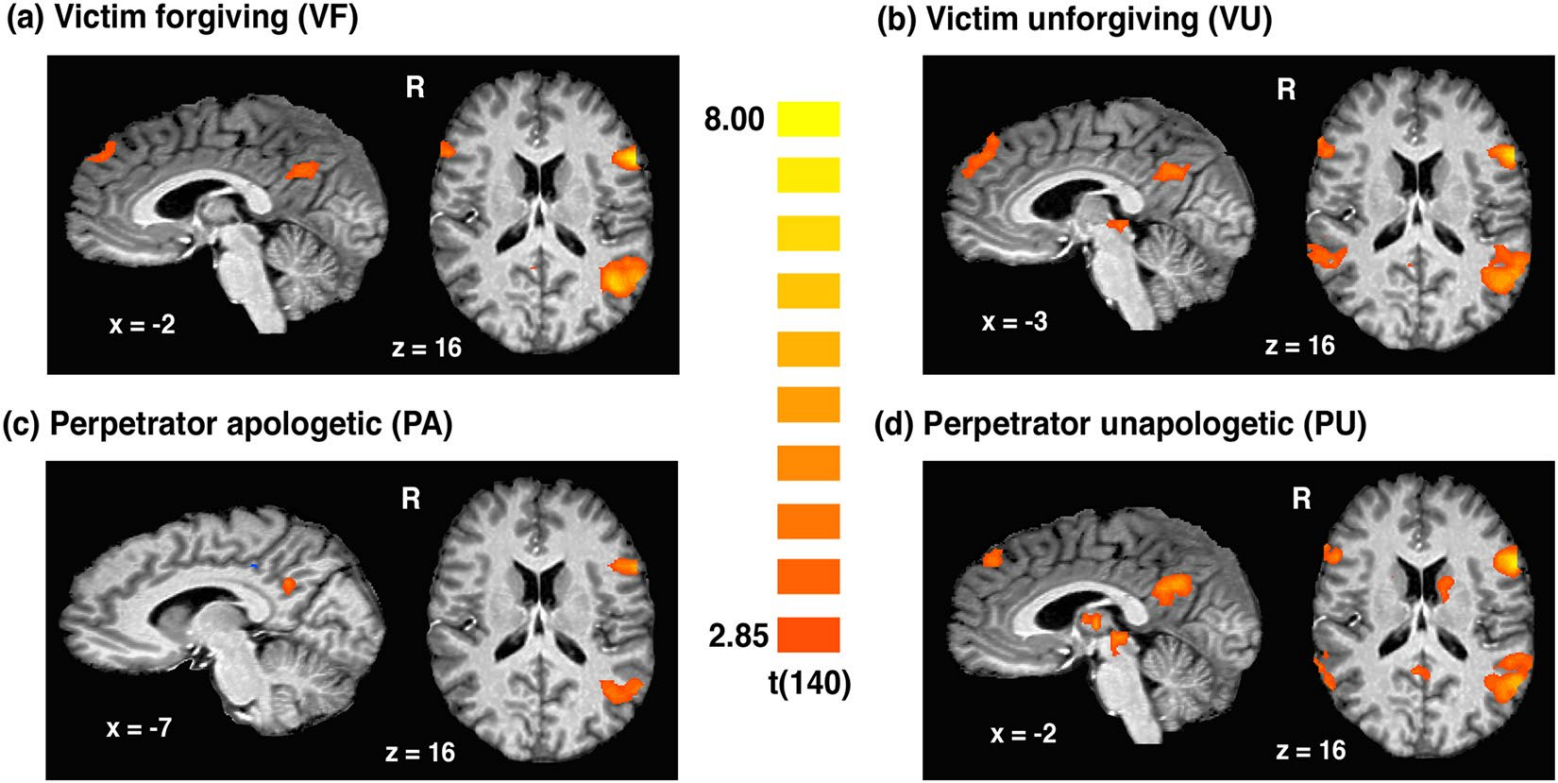
Whole-brain contrasts for the 4 conditions of interest (VF, VU, PA, and PU) against the neutral condition displayed on sagittal (left) and axial (right) sections in Talaraich space *(p* < 0.005 corrected for multiple comparisons using Monte Carlo cluster-level thresholding).

The contrasts for the 4 conditions of interest against the neutral condition revealed increased activity predominantly in areas associated with mentalizing (see Fig. 2, Supplementary Tables S5–S8). Specifically, all conditions showed heightened activity in the middle temporal gyrus, temporal pole, precuneus, and IFG. Heightened activity in TPJ, dmPFC and premotor cortex was additionally observed for the VF, VU, and PU conditions. Finally, the PU condition also showed significant activation in the periaqueductal gray (PAG), amygdala, and dorsal striatum.

#### Whole-brain main effect of participant group

Next, to examine group differences in empathic responding, we looked at the main effect of participant group. Here we observed increased activity in ventromedial prefrontal cortex (vmPFC), dACC, left dorsolateral prefrontal cortex (dlPFC), right pSTS, inferior parietal lobe, and thalamus (see Supplementary Table S9).

**Figure 3:**
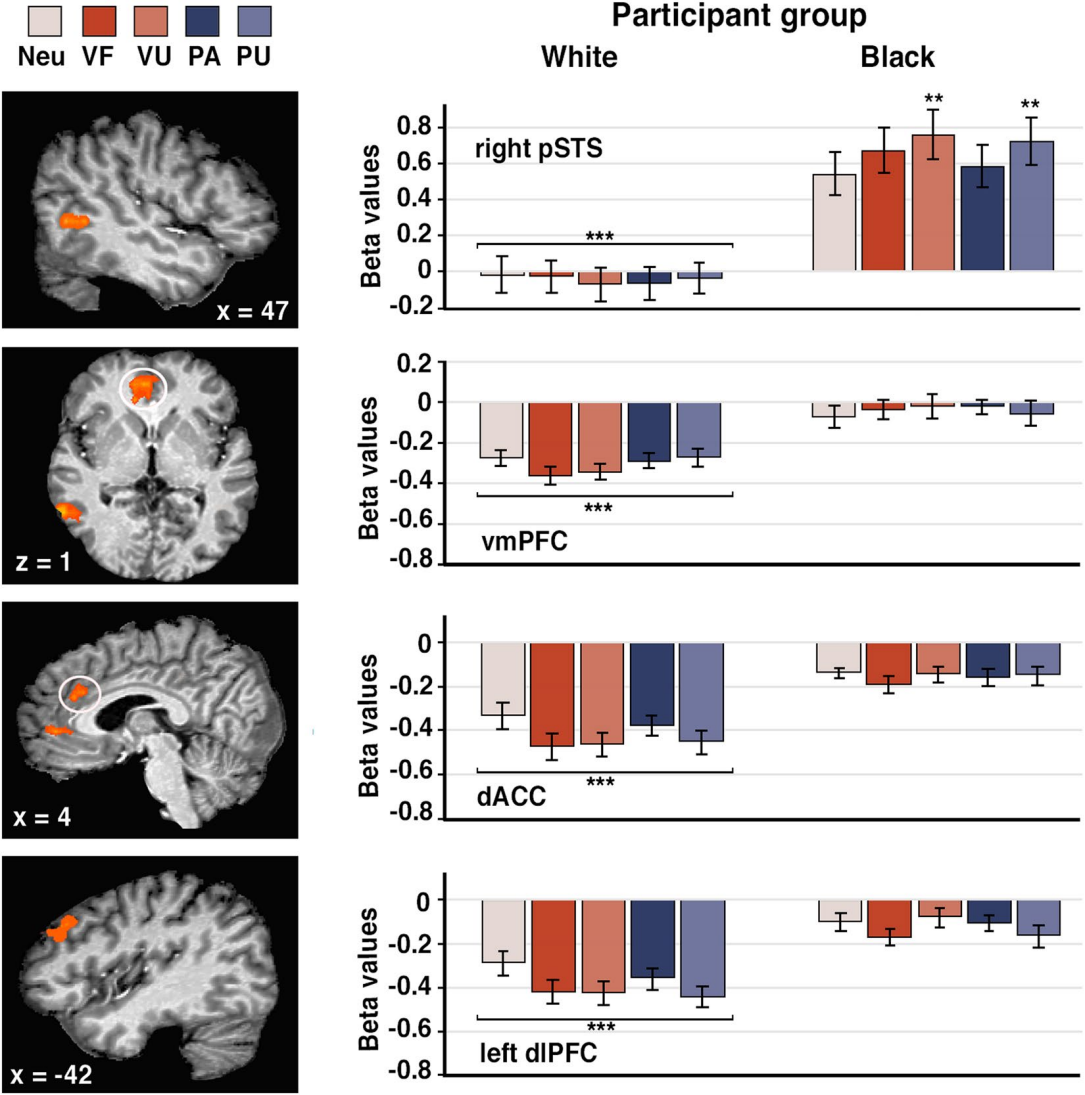
Regions showing a significant main effect of participant group (left panel). Activation in the right pSTS, vmPFC, dACC, and left dlPFC were significantly greater for Black compared to White individuals (*p* < 0.005 corrected for multiple comparisons using Monte Carlo cluster-level thresholding). Parameter estimates (betas) reflect the average signal intensity for each cluster for each condition (right panel). Error bars indicate standard error of the mean. dACC: dorsal anterior cingulate cortex, dlPFC: dorsolateral prefrontal cortex, pSTS: posterior superior temporal sulcus, vmPFC: ventromedial prefrontal cortex. **p < 0.01. ***p < 0.001.

Beta values extracted from these functionally defined ROIs and analyzed by 2-way participant group × condition ANOVAs confirmed significant main effects for participant group (*F*s > 21.50, *p*s < 0.001), such that activation in all areas were greater for Black compared to White participants (Fig. 3, Supplementary Table S10). We also found a significant interaction in the right pSTS (*F*(4, 136) = 4.03, *p* = 0.004), qualified by the fact that signal increase from neutral (Neu) to VU and Neu to PU conditions was greater for Black than White participants (*p*s < 0.01, *r*s > 0.44).

#### Independent ROI analyses

We assessed extracted beta values in ROIs using 2-way participant group × condition ANOVAs (Supplementary Table S11).

Affective empathy areas: No significant main effects of condition were observed for dACC or aINS. Consistent with the above, we found only a significant main effect of participant group in dACC (*F*(1, 34) = 6.90, *p* = 0.01), with activation greater for Black compared to White participants. No interaction effects were found.

**Figure 4:**
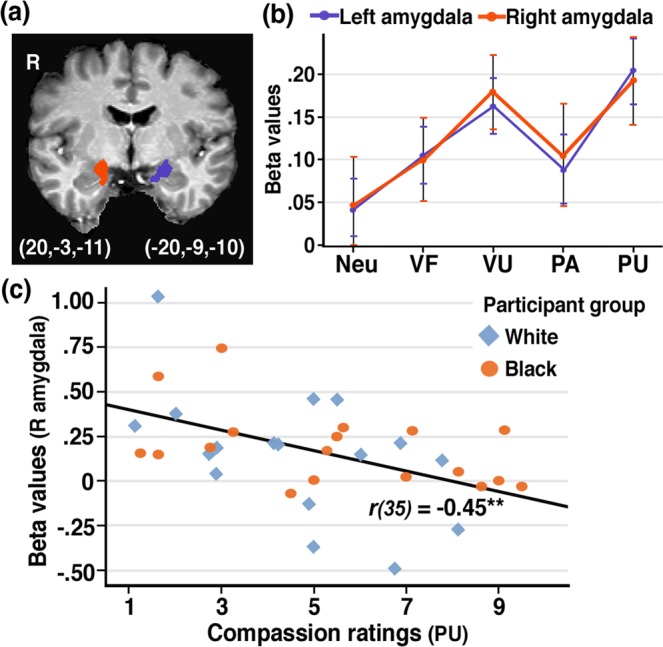
Amygdala reactivity. (**a**) Image shows ROI peak voxels defined based on independent data^29^. (**b**) Parameter estimates (betas) reflect the average signal intensity in the left and right amygdala in response to the various experimental conditions. (**c**) Higher activation in the right amygdala was associated with reduced compassion ratings in response to the PU condition across participants. Error bars indicate standard error of the mean. VF: victim forgiving, VU: victim unforgiving, PA: perpetrator apologetic, PU: perpetrator unapologetic. **p < 0.01.

ROI analyses for amygdala revealed a significant main effect of condition (*F*s > 4.00, *p*s < 0.005). Planned contrasts indicated that activation during the PU condition was significantly higher than activation during the Neu, VF, and PA (but not VU) conditions for both left (*p*s < 0.003, *r*s > 0.48) and right (*p*s < 0.02, *r*s > 0.38) amygdala (Fig. 4).

Mentalizing areas: We detected significant main effects of condition for the dmPFC, bilateral IFG, bilateral TPJ, and precuneus (*F*s > 5.0, *p*s < 0.001). Planned contrasts indicated that, for all areas, activation during the four conditions of interest was significantly higher than the Neu condition (*p*s < 0.05, *r*s > 0.32). In addition, activation during the PU condition was significantly higher than all other conditions for the left IFG (*p*s < 0.04, *r*s > 0.35), and higher than the PA condition for the right IFG, left and right TPJ, and precuneus (*p*s < 0.05, *r*s > 0.36). No other contrasts reached significance.

#### Brain-behavior correlations

Correlation data are presented in Supplementary Table S12.

Compassion: Across participants, higher right amygdala activation during the PU condition was associated with reduced compassion ratings in response to PU clips (*r*_*35(2-tailed)*_ = −0.45, *p* = 0.006) (Fig. 4). No significant correlations were observed for other affective empathy areas.

Regarding mentalizing areas, in Black participants, higher left TPJ activation during the VU condition was associated with reduced compassion ratings in response to VU clips (*r*_*18(2-tailed*_*)* = −0.53, *p* = 0.03). Similarly, higher left IFG activation during the VU and PU conditions were associated with reduced compassion ratings in response to VU and PU clips, respectively (*r*s > −0.50, *p*s < 0.05). No corresponding significant correlations were observed for White participants.

Social discrimination and early adversity: In White participants, everyday discrimination scores were associated with increased activation in the right amygdala in response to VU, PA and PU conditions (*r*s > 0.54, *p*s < 0.03), and left amygdala in response to VF and PA conditions (*r*s > 0.50, *p*s < 0.04). No significant correlations were observed for other affective empathy areas.

Regarding mentalizing areas, in Black participants, higher everyday discrimination scores were associated with increased activation in the left TPJ in response to the VU condition (*r*_*18(2-tailed*_*)* = 0.67, *p* = 0.003). Furthermore, higher everyday discrimination scores were associated with increased activation in the left IFG in response to VU and PU conditions (*r*_*18(2-tailed)*_ = 0.64, *p* = 0.005 and *r*_*18(2-tailed)*_ = 0.56, *p* = 0.02, respectively). Similarly, higher CTQ emotional abuse scores were associated with increased activation in the left TPJ in response to all four conditions (*rs* > 0.57, *p* < 0.02). Again, no corresponding significant correlations were observed for White participants.

**Figure 5:**
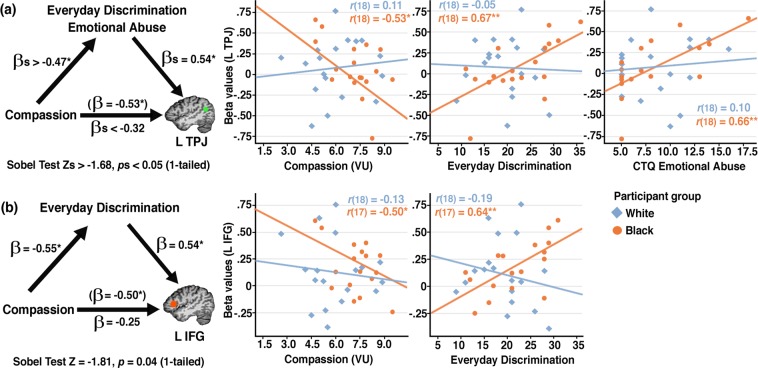
Mediation analysis for the victim unforgiving (VU) condition. (**a**) Both social discrimination (Everyday Discrimination scores) and early adversity (CTQ Emotional Abuse scores) mediated the negative relationship between reported compassion and left TPJ activation (*R*^2^ > 0.48, *p*s < 0.01). (**b**) Social discrimination also mediated the negative relationship between reported compassion and left IFG activation (*R*^2^ = 0.46, *p* = 0.01). Sobel tests showed that, in each case, including the mediator in the model significantly reduced the effect of compassion on brain activation. Scatter plots show the correlations (Pearson’s *r*, 2-tailed) between b*r*ain activation in the left TPJ/IFG and reported compassion and social adversity for Black and White participants, respectively. Brain images show corresponding ROIs based on independent data^61^. *p < 0.05. **p < 0.01.

To explain the mechanism underlying some of these observed relationships, we conducted exploratory mediation analyses (Fig. 5)^54^. These analyses suggested that for Black participants, everyday discrimination and CTQ emotional abuse, two highly correlated constructs (*r* = 0.78, *p* < 0.001), each mediated the negative relationship between compassion ratings and activation in the left TPJ for the VU condition: Everyday discrimination and CTQ emotional abuse scores predicted neural activity while controlling for compassion ratings (βs = 0.54, *p*s < 0.03), but rendered nonsignificant the negative relationship between compassion ratings and left TPJ activation (βs < −0.32, *p*s > 0.12), indicating full mediation (Sobel test *p*s < 0.05). Everyday discrimination also fully mediated the negative relationship between compassion ratings and activation in the left IFG for the VU condition (β = 0.54, *p* = 0.02, while controlling for compassion ratings). While these effects were strongest for the VU condition, a similar mediation effect in the left IFG for everyday discrimination was also observed for the PU condition (see Supplementary Table S13).

## Discussion

Whereas sustained neural effects of early adversity are well documented, our understanding of how enduring forms of social discrimination impact brain functioning remains poor. The present research investigated the effects of early adversity and social discrimination on neural response when White and Black South Africans empathized with apartheid victims and perpetrators. Overall, the findings suggest that Black compared to White participants responded with heightened empathic sensitivity. In addition to greater reported compassion when witnessing victim and perpetrator testimonies, their neural responses were characterized by heightened activation in areas associated with generation of affective meaning and salience (vmPFC, dACC), as well as with mental state representation (pSTS). Greater experiences of social adversity (early adversity and social discrimination) were associated with reduced reported compassion across participants. In White participants, social discrimination (primarily because of income level/weight/gender) was associated with undifferentiated amygdala reactivity. By contrast, in Black participants, social discrimination (primarily because of race) and early adversity mediated the negative relationships between brain activation (TPJ, IFG) and compassion for unforgiving and unapologetic individuals. These findings suggest that areas involved in stress-related circuitry and social information processing are impacted differentially in terms of empathic responding in people who have endured cumulative experiences of social adversity.

Consistent with our first hypothesis, the findings confirmed that empathy in response to complex mental states is associated more with effortful cognitive perspective taking (i.e., heightened activation in temporal areas, precuneus, IFG, and dmPFC) than with affective empathy mechanisms. Here, the complexity of targets’ mental states, combined with the age of participants, and the explicit instruction to imagine how victims/perpetrators experienced their situation, likely facilitated cognitive perspective taking^28,55^. Likewise, lack of significant dACC and aINS activation might be explained by the nature of our task: previous empathy research often made use of highly salient, brief stimuli that are associated with a detecting/orienting response not specific to pain perception^56,57^. Nevertheless, recent research suggests that empathic accuracy is more dependent on cognitive perspective taking than affective sharing^58^.

Interestingly, viewing of unapologetic perpetrators was associated with (i) significant amygdala and PAG activation (whole-brain analysis), (ii) the highest left IFG activation (ROI analysis), and (iii) behaviorally, with the lowest compassion and highest moral indignation ratings. Consistent with these findings, elevated right amygdala activation during the PU condition was associated with reduced compassion ratings across participants. Because the amygdala is sensitive to negative, threat-related stimuli^59^, and involved in evaluating high-level social information, such as trustworthiness^60^, these results are consistent with previous research.

The IFG has been associated with a host of cognitive capacities, most notably mentalizing^61,62^, but also emotion regulation^63^ and understanding^64^. However, the IFG likely plays a more general role in cognition^65^. Tops and Boksem describe the importance of the IFG in ventral corticolimbic control pathways that manage attention and behavior in situations with low predictability^66,67^. Accordingly, the IFG supports a reactive kind of behavioral control that is engaged when attentional focus is on urgent events discrepant with expectancies, thus recruiting greater evaluative processes to guide behavior. While this interpretation relies on reverse inference, we believe that, using a likelihoodist approach, it is more probable than interpretations focusing on emotion understanding^65,68^.

Consistent with our second hypothesis, we observed significant group differences. Black compared to White participants responded with greater self-reported compassion (and moral indignation) to the video clips, and at a neural level their responses were characterized by heightened activation in dACC, thalamus, vmPFC, dlPFC and pSTS. As detailed below, one interpretation of these findings is increased empathic sensitivity, but also greater subjective valuation and overt mental state representation for Black versus White participants.

The dACC and thalamus form part of the paralimbic salience network, which directs attention to events of personal importance (both internal and external), thereby determining selection for in-depth processing^50,69^. The dACC, in particular, may play an important role in indexing socially pertinent information and integrating motivationally relevant information with downstream physiological reactions^70,71^. Hence, the dACC is also crucial for empathic sensitivity—increased arousal when witnessing another in distress may be associated with more physiological signals to help interpret the target’s emotional state and potentially promote empathic concern^17^. By comparison, White participants’ responses may be more indicative of emotional blunting. A previous fMRI study found that White participants’ reaction of guilt, and especially shame, in response to Truth and Reconciliation Commission (TRC) footage were associated with reduced activation in areas associated with affective empathy^31^. Likewise, enhanced guilt and shame reported here by White participants might have promoted a more egocentric focus and disengagement from the shame-inducing stimuli^72,73^.

The vmPFC has reciprocal connections with the amygdala and hypothalamus and has consistently been implicated in the regulation of emotional responsiveness and empathic concern^24,74–76^. Recent theorizing suggests the vmPFC is a core area encoding subjective value of social and non-social stimuli in a context and goal-dependent manner^77,78^. The vmPFC thus appears unnecessary for simple forms of affectivity, but essential for the generation of affective *meaning* to coordinate appropriate physiological emotional responses and decision-making. Generating affective meaning includes representing the affective qualities of an event, its value, similar past situations, and potential outcomes^79^. Thus, increased activity in vmPFC for Back participants when viewing the clips might imply heightened meaning-making/valuation, and by extension, empathic concern.

The pSTS appears involved differentially in the visual analysis and interpretation of socially salient cues, such as bodily motion and facial emotion, in evaluating goal-directed action, and in processing overt (versus covert) mental states^80–82^. In our task, facial expressions and bodily motion were typically more exaggerated in VU and PU conditions, which portrayed more anger/resentment than the other conditions and might explain the heightened pSTS activation. Importantly, heightened mental state representation for these conditions was not necessarily associated with heightened compassion, as the PU condition was associated with the lowest compassion and highest moral indignation ratings. Indeed, previous research suggests the mentalizing network activates robustly both in the presence and absence of harmful mental states^83,84^.

The above group differences can also be interpreted to support the hypothesis that social marginalization cumulatively impacts the neural response in ACC, resulting in altered salience attribution and hypervigilance with regard to race-related discrepancies^47,49^. Indeed, the high reported moral indignation and concomitant dlPFC activation suggest engagement of the appraisal system to self-regulate^48^. During post-experiment interviews, several Black participants reported that, in addition to “feeling sorry”, the video clips triggered anger for them at the lack of social justice and change since 1994. In this regard, research has shown that high ingroup empathy can trigger hostility toward an outgroup perceived to be the source of one’s suffering^85,86^. The present material likely resonated with both apartheid memory and its legacies in the present, initiating other self-reflective processes. Thus, in the current context, enhanced dACC activation might also reflect affective dissonance^87,88^.

In sum, while Black participants may experience heightened empathic sensitivity to emotionally charged material in the moment, frequently needing to exert emotional control could tax executive processes, potentially leaving individuals feeling distressed and vulnerable to subsequent stressors^48,89^.

Our third hypothesis concerned individual differences. The present finding that social discrimination and early adversity across participants were associated with reduced reported compassion is consistent with literature. Previous reports cite early adversity as being significantly associated with impaired empathy^19–21^, including difficulty caring for or taking the perspectives of others. Similarly, research suggests that chronic experiences of discrimination are typically experienced as stressful, and may undermine empathy^10,90^. Both anxious emotions, that enhance egocentrism, and heightened cognitive load have been shown to impact empathy-related processing adversely^91,92^.

Social adversity was also associated with functional alterations in areas associated with stress reactivity and social information processing. Specifically, in White participants, social discrimination was associated with heightened bilateral amygdala reactivity in response to all conditions. This finding is consistent with literature suggesting that perceptions of negative social treatment is associated with increased emotional reactivity and reduced specificity in response to socially salient stimuli^46,49,93^. Heightened amygdala reactivity has been associated with higher levels of current psychological stress as well as with stress-related mental disorders^94–96^.

By comparison, in Black participants, social adversity was associated with heightened activation in the left TPJ and IFG. Moreover, childhood emotional abuse, and especially social discrimination, mediated the negative relationships between reported compassion and brain activation in these structures for the VU and PU conditions. Whereas discrimination-associated altered responses in White participants were thus indicative of emotional reactivity, the findings for Black participants are consistent with the hypothesis that discrimination-associated heightened activity in higher-order social information processing areas allows more fine-tuned distinctions between conditions, and hence decreased compassion specifically towards unforgiving and unapologetic individuals.

Elevated activity in social information processing areas in adults who experienced early adversity have previously been proposed to reflect strategies that compensate for deficits in emotional empathy by recruiting more effortful cognitive empathy^44,45^. However, in the present context, heightened activity in TPJ and IFG were associated with appropriately reduced compassion. A more likely explanation, then, is that these responses reflect increased identification and representation of negative social cues^43^. Early detection of threatening stimuli, particularly angry emotions, is adaptive in contexts of childhood adversity^97^. Likewise, individuals who regularly experience race-based rejection are more vigilant with regard to environmental threats^23^.

The TPJ and IFG (together with the STS and inferior parietal lobe) form a large part of the ventral attention network, which is sensitive to behaviorally relevant, novel events^98,99^. According to predictive and reactive control systems theory (PARCS), response biases in this network may result from long-term exposure to unpredictable environments, such as those associated with inconsistent parenting and trauma, and may suggest a more reactive kind of behavioral control guided by momentary environmental stimuli^66,67,100^. If sustained, this mode of behavioral control might predispose the individual to anxious anticipation of negative events and potentially mood disturbances^100,101^. Yet in the current context, response biases were not generalized, but elevated in response to the more emotionally charged and unpredictable conditions, potentially reflecting adaptive responses to meet current challenges.

Given that both early adversity (emotional abuse) and social discrimination were associated with elevated activation in areas associated with reactive control, it should be noted that these constructs were highly correlated and explained a common variance regarding brain activation responses. As noted, this might be due to early adversity and social discrimination often coinciding in marginalized populations, but also because those who suffered early experiences of abuse might be more vulnerable to social mistreatment^102,103^. In the absence of longitudinal data, any causal explanations remain speculative.

A key question is why, given the comparable levels of reported social adversity across participant groups, responses differed at a neural level. For early adversity, the macro context of White participants is perhaps more protective in terms of resources than those of economically marginalized Black participants, possibly buffering the negative effects of maltreatment^104^. For social discrimination, however, the explanation likely involves qualitative differences in perceived discrimination: race for Black participants; income level, gender, and weight for White participants. Research suggests that appraisals of negative social treatment and coping mechanisms vary as a function of the type of discrimination^102,105^. Experiences of negative social treatment may furthermore be more distressing for disadvantaged than advantaged groups, because their attributions of prejudice are likely to be more internalized and stable over time^106,107^. On the other hand, factors such as income level and weight are perceived to be under personal control. Group memberships based on such characteristics may be associated with greater self-stigma, which is more isolating and related to poorer well-being than memberships based on factors beyond personal control^8,108^. Indeed, pervasive discrimination against members of disadvantaged groups may result in strong connections with fellow group members, which serves as a coping mechanism to counter psychological distress^109,110^. What seems to matter, then, is whether group identity has positive or negative value for the individual^6^.

A few limitations of this study deserve emphasis. First, our design does not allow causal inferences to be made from the observed associations of social adversity. While only correlations with large effect sizes were reported, they should be interpreted with caution due to the relatively small sample size. Our data were obtained from a non-clinical sample and hence cannot prove increased risk towards subsequent socioaffective disturbances in either White or Black participants. The data also do not allow us to comment on whether alterations in functional activity are associated with enduring structural changes.

Second, because we relied partially on reverse inference to infer psychological processes from observed patterns of brain activation, the inductive validity of these inferences may be questioned^111^. Future research could minimize such concerns by recasting reverse inferences in likelihoodist terms where applicable, as we have (i.e., deciding which of two competing hypotheses is best supported by the data). This approach has been proposed to circumvent the issues associated with reverse inference^68^. In addition, future enquiries would benefit from including neuroimaging tasks with interpretable behavioral evidence that manipulate specific psychological processes, such as subjective valuation, mental state representation, and self-regulation, which would validate inferences by enhancing the construct validity of neural responses beyond our passive viewing paradigm^112^.

Finally, because marginalization is not restricted to discrete experiences, but manifests in the pervasive, ongoing, systematic, structural violence of the entire social space, it may be subject to underreporting^49^. Furthermore, individual differences in attributing negative experiences to discrimination (e.g., vigilance versus minimization bias)^113^, may limit replication of our results. It is thus possible that elevations in amygdala, TPJ, and IFG activity reflect innate response biases, rather than social discrimination per se.

In conclusion, our results elucidate the neural effects of social adversity in terms of functional changes in cognitive and affective empathy mechanisms. Whereas social discrimination in White participants was associated with greater amygdala reactivity suggesting altered stress responsivity, social adversity in Black participants was associated with increased activation in mentalizing and social information processing structures and decreased compassion to emotionally provocative conditions. These data extend the literature in two important ways: First, it shows that experiences of social discrimination have comparable associations at the neural level as other types of psychosocial stress. Second, it provides an initial framework for understanding how empathy might be modulated in those who endure cumulative experiences of social discrimination, with the nature of the stigma likely contributing significantly to altered neural response patterns. Beyond these more direct associations with social adversity, however, the present research also informs our understanding of the mechanisms that might impact empathic responding in members of high-status groups (e.g., emotional blunting) when they bear witness to trauma testimonies. More detailed investigation of these processes may ultimately be useful in facilitating psychological repair in the wake of historical trauma, and in moving towards more socially just and equal societies.

## Methods

### Participants

Thirty-six South Africans, 18 who identified as Black African (10 female, *M* = 40.28 years, *SD* = 4.17), and 18 who identified as White (8 female, *M* = 40.83 years, *SD* = 6.07), recruited through local newspaper advertisements, completed all study procedures and received ZAR200 compensation. All participants lived in South Africa during apartheid (prior to 1994) and obtained Grade 12 as a minimum level of education (Black African: *M* = 16.28 years, *SD* = 2.30; White: *M* = 16.22 years, *SD* = 3.04). Participants were without previously diagnosed neurological, cardiovascular, or psychiatric disorders, and none were clinically depressed^114^.

All participants provided informed consent. The study was approved by the University of Cape Town’s Human Research Ethics Committee and all procedures were carried out according to these guidelines.

### Questionnaire measures

#### Social discrimination

To assess relatively minor experiences of unfair treatment that contribute to a type of chronic stress, the Everyday Discrimination Scale was used^115^. This 9-item scale assesses the frequency of different forms of social mistreatment from 1 (*Never*) to 6 (*Almost Everyday*), and has been shown to have good internal validity in the South African context^116^. If participants experienced discrimination “*A few times a year*” or more frequently, they were asked to indicate what they considered the main reason(s) for these experiences were (e.g., race, gender, physical appearance, or income level).

#### Early adversity

The Childhood Trauma Questionnaire Short-Form (CTQ-SF) was used to assess the severity of child maltreatment^117^. This 25-item retrospective measure is used extensively in peer-reviewed research and has good validity across clinical and nonclinical populations^4^. It records the frequency of physical neglect, emotional neglect, physical abuse, sexual abuse, and emotional abuse when participants “were growing up”, ranging from 1 (*Never True*) to 5 (*Very Often True*).

Questionnaire measures were completed several weeks before scanning during an online survey.

### Stimuli

Stimuli consisted of 50 short 6–9 s video clips (352 pixels × 288 pixels) featuring Black African or White individuals either as victims expressing (i) forgiveness (VF) or (ii) unforgiveness (VU), as perpetrators who are (iii) apologetic (PA) or (iv) unapologetic (PU), or as (v) individuals expressing neutral views (Neu). The verbal content of the clips were sourced from actual TRC hearings and related documentaries but were reproduced (standardized) for the purposes of this study using actors. Neutral clips included statements about everyday events unrelated to the TRC. Video clips were recorded with a digital color camcorder from a frontal view that included the actor’s whole head and parts of the shoulders. The actors were seated in front of a white background and were instructed to direct their gaze at a point about 30 cm to the left of the camcorder to avoid direct eye contact, giving the impression of being in conversation with an interviewer. All clips were validated by a mixed-race sample of 57 volunteers in an independent behavioral experiment (see Supplementary Material). During scanning, clips were presented three at a time in blocked format, separated by a 500 ms scrambled static grey image, and superseded by a 3 s title indicating the nature of the block (e.g., Victim Forgiving). Clips within each block were randomized and included at least one Black and one White individual. No clip was repeated more than once.

### Procedure

Participants received standardized instructions about the purpose of the study, namely to understand how the human brain responds to others’ distress. Specifically, they learned that they would watch short video clips of victims and perpetrators from the TRC hearings, who were interviewed again after the hearings and asked how they felt about past events. It was highlighted that victims were forgiving or unforgiving, whereas perpetrators were apologetic or unapologetic about their crimes. They were also told that some individuals expressed views unrelated to the TRC (i.e., neutral clips). To ensure participants understood the context of the TRC clips, they were shown a 2 min video about the TRC, as well as an example of a clip from each condition. They were instructed to empathize with individuals in each clip and to imagine how they felt in their situation.

In the scanner, participants viewed stimuli through a mirror system mounted to the head coil, which was displayed with *E-Prime* software, version 2.0 (Psychology Software Tools, Inc.). Participants first underwent a structural scan, followed by three runs of stimuli counterbalanced across participants. Each run consisted of 10 blocks (2 per condition), with the order of conditions randomized within a half run. The interval between blocks lasted 10 s during which participants fixated at a central cross.

### Post-scan emotion ratings

After the scan, participants reported how much they felt distressed (personal distress), sorry (compassion/empathic concern), angry (moral indignation), guilty, and ashamed in response to each clip on separate 1 (*not at all*) to 9 (*extremely*) visual analog scales (VAS). Participants were then briefly interviewed about their experiences in the scanner and debriefed. They were also offered the opportunity to see a counsellor, should they wish to further discuss their experiences.

### fMRI Image acquisition and data analysis

MRI data were acquired on a 3T Allegra system (Siemens, Erlangen, Germany). The high-resolution anatomical scan was acquired with a *T*_1_-weighted sequence (3D mprage, TR/TE = 2530/6.5 ms). Functional images covering the whole brain were acquired with a *T*_2_*-weighted echo-planar (EPI) imaging sequence using blood-oxygenation-level-dependent (BOLD) contrast (TR/TE = 2000/30 ms, slice thickness = 3 mm, gap = 0.9 mm, flip angle = 90°, field of view = 240 × 240 mm). The first four volumes of each run were discarded to allow for *T*_1_ equilibration effects.

All fMRI analyses were performed using Brain Voyager QX, version 2.8 (Brain Innovation, Maastricht, Netherlands). Preprocessing of images included correction for slice acquisition times and linear trends, temporal filtering with a high-pass filter of 2 cycles/point, and motion-correction relative to the first volume of each run with trilinear/sinc interpolation. No run exceeded 3 mm displacement/3.0° rotation. Participants’ functional data sets were co-registered with their structural MRI and spatially normalized to Talaraich space.

Whole-brain group analyses were performed with a random effects analysis of variance using the general linear model (GLM) with predictors corresponding to known experimental blocks convolved by the standard haemodynamic response function. We defined predictors for the 5 conditions: VF, VU, PA, PU, and Neu. The six z-transformed motion correction parameters were added as predictors of no interest to reduce motion artifacts.

The resulting estimated beta values were entered into a second-level two-factor mixed factorial ANOVA, with the between-subjects factor participant group (Black vs. White), and the within-subjects factor condition (5 levels). To assess specific condition effects, we contrasted each of the four conditions of interest against the neutral condition. In addition, we evaluated the main effect of participant group. All whole-brain results are reported at *P* < 0.005, corrected for multiple comparisons using the Monte Carlo cluster threshold estimation simulation tool implemented in Brain Voyager running1000 iterations^118^. To further explore these results, region of interest (ROI) analyses were performed for the activated clusters that emerged in the whole-brain main effect of participant group analysis (shown in Supplementary Table S9). Random effects analysis of variance was performed on the average signal in each cluster for each participant using the GLM described above. Beta values generated by this analysis (reflecting the mean percent signal change for each condition) were analyzed by 2-way ANOVA.

To enhance the power of our analyses and reduce the Type-I error rate by performing fewer statistical tests^119^, we defined hypothesis-driven independent ROIs. Areas most consistently implicated in experience sharing (affective empathy: dACC, aINS) and mentalizing (cognitive empathy: dmPFC, TPJ, precuneus, and IFG), respectively, were defined based on recent meta-analyses (see Supplementary Table S2 for coordinates)^29,61^. In addition, we selected ROIs for the amygdala, because previous studies investigating the neural sequelae of early adversity have consistently reported heightened amygdala reactivity during emotional processing^43^. All regions were defined as spheres with 8 mm radiuses centered at the peak voxel in each cluster, except for the amygdala, where spheres were defined with 5 mm radiuses. ROI analyses for these independently selected regions were conducted as described above, by computing beta estimates representing the average percent signal change for each region and analyzing by 2-way ANOVA.

To examine relationships between social adversity and brain activity, zero-order correlations between ROI beta values for our 4 conditions of interest and behavioral data (childhood maltreatment, everyday discrimination and compassion scores) were inspected. ROIs included in these analyses were those associated with affective and cognitive empathy, as well as the amygdala. To limit the number of correlations^120^, only correlation coefficients reflecting large effect sizes (*r* ≥ ±0.50), or those significant at the 1% level (i.e., *p* < 0.01) were interpreted and further inspected using 95% confidence intervals (CIs) derived through bootstrapping.

## Supplementary information

Supplementary Material

## Data Availability

The datasets generated during and analyzed during the current study are available from the corresponding author on reasonable request.
