# Supplementary material for: Effects of early adversity and social discrimination on empathy for complex mental states: An fMRI investigation
Source: Sci Rep. 2019 Sep 10;9:12959. doi: 10.1038/s41598-019-49298-4 (PMC6737126; doi:10.1038/s41598-019-49298-4)
Supplement: Supplementary file 1 — Supplementary Material [file 41598_2019_49298_MOESM1_ESM.docx]

**Effects of early adversity and social discrimination on empathy for complex mental states: An fMRI investigation**

Melike M. Fourie, Dan J. Stein, Mark Solms, Pumla Gobodo-Madikizela, & Jean Decety

# SUPPLEMENTARY MATERIAL

**Supplementary Methods**

***Stimulus Validation Study***

To ensure that video clips generated for the purposes of this study were believable and of comparable emotional intensity, we conducted a stimulus validation study.

A sample of 57 individuals (45 female; Black African: *n* = 20, White: *n* = 23, Other (Coloured and Indian): *n* = 14), none of whom participated in the main study, evaluated the filmed video clips. The stimuli for this validation study consisted of 95 video clips: 17 victim forgiving (VF), 21 victim unforgiving (VU), 14 perpetrator apologetic (PA), 21 perpetrator unapologetic (PU), and 22 neutral (Neu) clips. All clips were rated on visual analog scales for their believability (*How genuine is this person?*), ranging from 1 (*Not at All*) to 9 (*Very Much*). In addition, all clips were rated along the empathic dimensions of affective arousal (*How aroused did you feel in response to this clip?*), perspective taking (*How intense is this person’s distress?*), and empathic concern (*How sorry did you feel for this person?*), from 1 (*Not at All*) to 9 (*Very Much*).

Video clips were edited to be 6-9 s in duration and featured either a Black or White individual. Clips of perpetrators portrayed individuals who had perpetrated violence on both sides of the political apartheid conflict (e.g., members of the state security police, Black activists, and soldiers in military units), and who responded in either an apologetic (e.g., showing remorse or asking for forgiveness) or unapologetic fashion (showing no remorse) regarding their crimes. Unapologetic perpetrators tended to justify/absolve themselves by arguing that their actions were politically motivated. By contrast, clips of victims portrayed individuals who had lost a loved one as a result of these violent crimes during the apartheid conflict, and who responded in either a forgiving or unforgiving manner. Neutral clips included statements about everyday events that were not emotionally charged. Examples of stimuli for each condition are displayed below:

*Victim Forgiving:* I felt the right message was no bitterness, not seeking revenge, and offering forgiveness, that very night, knowing my wife was dead.

*Victim Unforgiving:* I’m not going to absolve him, you know...if he wants to feel lighter I’m not the person who’s going to do that now⎯I refuse to do that.

*Perpetrator Apologetic*: I know that I have done wrong, that I have done evil things. I want to say to you as the parents of those children, I ask your forgiveness from the bottom of my heart.

*Perpetrator Unapologetic*: I don’t think I have to say I’m sorry, and I’m not going to say it...for what I did. Because I was doing my job, and I thought it was right.

*Neutral*: What I am most concerned about is the lack of parking space on upper campus, and especially on middle campus, during the lunch hour.

Based on participants’ responses, we selected the 10 clips per condition (5 Black, 5 White) that scored the highest in terms of believability and their ratings on the various empathy dimensions (see Table S1). In each condition, the Black and White clips were also matched for sex.

Table S1

*Participant Ratings (N = 57) for Selected Video Clips (n = 10) in Each Condition*

| Condition | Ratings | | | |
| --- | --- | --- | --- | --- |
|  | Believability/ authenticity | Arousal value | Distress intensity | Empathic concern |
| Victim Forgiving (VF) | 6.67 (1.08) | 5.25 (1.39) | 5.83 (1.10) | 5.91 (1.25) |
| Victim Unforgiving (VU) | 7.10 (1.27) | 5.99 (1.55) | 7.45 (1.12) | 6.27 (1.39) |
| Perpetrator Apologetic (PA) | 6.58 (1.03) | 5.39 (1.33) | 5.83 (1.16) | 5.31 (1.36) |
| Perpetrator Unapologetic (PU) | 6.46 (1.19) | 5.94 (1.62) | 4.51 (1.20) | 3.88 (1.79) |
| Neutral (Neu) | 5.99 (1.66) | 2.70 (1.46) | 2.08 (1.30) | 2.04 (1.22) |

*Note.* Data presented are means, with standard deviations in parentheses. Ratings ranged from 1 (*not at all*) to 9 (*extremely*).

As can be seen from Table S1, all clips were rated as significantly above average on believability, *t*(56)s > 4.5, *p*s < 0.001. In fact, most participants did not suspect that any of the clips were filmed by actors. Further, and in line with our expectations, whereas neutral clips were high in believability, they were rated as significantly lower than all other clips in terms of the empathy dimensions assessed (*p*s < 0.001).

Three separate 3 (participant group: Black, White, Other) x 5 (condition: Neu, VF, VU, PA, PU) mixed factorial ANOVAs further assessed participants’ responses in terms of affective arousal, distress intensity, and empathic concern. In terms of affective arousal, participants rated the arousal they experienced in response to the VU and PU clips as significantly higher than that of the VF and PA clips (*p*s < 0.001), while the arousal they experienced in response to the VF and PA clips was significantly higher than that of the Neu clips (*p*s < .001). Because the magnitude of actual differences in arousal ratings between our 4 conditions of interest were small (ranging from 5.25 to 5.94), we felt confident that these conditions were well matched in terms of orienting participants’ attention. Furthermore, there was no significant effect for participant group, and no significant interaction (*p*s > 0.15), suggesting that the different racial groups’ responses were similar. We therefore proceeded to employ these TRC clips in the fMRI paradigm.

We anticipated that our 4 conditions of interest would differ more markedly in terms of the perceived intensity of distress of the victim/perpetrator and empathic concern ratings, however. In terms of the perceived intensity of distress, the highest levels of perceived distress were reported for the VU compared to the other clips (*p*s < 0.001), while the perceived distress reported for the VF and PA clips were significantly higher than that of the PU clips (*p*s < 0.001), and the perceived distress of the PU clips was significantly higher than that of the Neu clips (*p* < 0.001). There was no significant effect for participant group, and no significant interaction effect (*p*s > 0.35). Finally, ratings for empathic concern followed the same pattern of response as that of perceived intensity of distress, except that empathic concern for the VF clips was also higher than that of the PA clips (*p* < 0.001), thus VU > VF > PA > PU > Neu. Again, there was no significant effect observed for participant group, and no significant interaction effects (*p*s > 0.54).

***ROI Analysis***

Table S2 shows information regarding the ROIs employed in fMRI data analysis. Coordinates previously reported in MNI space were converted to Talaraich space using the “MNI to Talaraich” conversion function implemented in GingerALE.

Table S2

*Regions of Interest (ROIs)*

|  |  | TAL Coordinates | | |  |
| --- | --- | --- | --- | --- | --- |
| Region | Hem. | *x* | *y* | *z* | Source |
|  |  |  |  |  |  |
| dACC | L/R | -3 | 16 | 42 | (Lamm et al., 2011) |
| aINS | R | 35 | 20 | 3 | (Lamm et al., 2011) |
|  | L | -38 | 19 | 5 | (Lamm et al., 2011) |
| Amygdala | R | 20 | -3 | -11 | (Lamm et al., 2011) |
|  | L | -20 | -9 | -10 | (Lamm et al., 2011) |
| dmPFC | L/R | -1 | 54 | 33 | (Schurz et al., 2014) |
| TPJ | R | 56 | -56 | 18 | (Schurz et al., 2014) |
|  | L | -53 | -59 | 20 | (Schurz et al., 2014) |
| Precuneus | L/R | 4 | -55 | 34 | (Schurz et al., 2014) |
| IFG | R | 44 | 20 | 12 | (Schurz et al., 2014) |
|  | L | -46 | 22 | 8 | (Schurz et al., 2014) |
|  |  |  |  |  |  |

*Note.* Talaraich coordinates refer to the peak of each brain region**.**

aINS = anterior insula; dACC = dorsal anterior cingulate cortex; dmPFC = dorsomedial prefrontal cortex; IFG = Inferior frontal gyrus; TPJ = temporoparietal junction

**Supplementary Results**

***Behavioral Data: Emotion Ratings***

Participant group x condition mixed factorial ANOVA statistics for each emotion rating are presented in Table S3.

Table S3

*ANOVA Summary Table for Each Emotion Rating*

| Source | *df* | MS | *F* | *p* | Effect size |
| --- | --- | --- | --- | --- | --- |
|  |  |  |  |  |  |
| Compassion |  |  |  |  |  |
| Condition (A) | 2.06 | 38.47 | 14.15 | < 0.000*** | 0.30 |
| Participant group (B) | 1 | 13.61 | 7.05 | 0.01** | 0.18 |
| A x B | 2.06 | 1.24 | 0.67 | 0.52 | 0.02 |
| Moral indignation |  |  |  |  |  |
| Condition (A) | 2.18 | 48.82 | 15.26 | < 0.000*** | 0.32 |
| Participant group (B) | 1 | 7.58 | 4.94 | 0.03* | 0.13 |
| A x B | 2.18 | 0.66 | 0.21 | 0.83 | 0.01 |
| Personal distress |  |  |  |  |  |
| Condition (A) | 3 | 4.08 | 3.89 | 0.01** | 0.10 |
| Participant group (B) | 1 | 2.40 | 1.05 | 0.31 | 0.03 |
| A x B | 3 | 0.86 | 0.82 | 0.48 | 0.02 |
| Guilt |  |  |  |  |  |
| Condition (A) | 2.11 | 2.59 | 2.44 | 0.09 | 0.07 |
| Participant group (B) | 1 | 0.00 | 0.00 | 0.98 | 0.00 |
| A x B | 2.11 | 4.69 | 4.41 | 0.01** | 0.12 |
| Shame |  |  |  |  |  |
| Condition (A) | 2.15 | .75 | 0.33 | 0.80 | 0.01 |
| Participant group (B) | 1 | 7.99 | 3.24 | 0.08 | 0.09 |
| A x B | 2.15 | 14.30 | 6.24 | 0.003** | 0.16 |
|  |  |  |  |  |  |

*Note*. MS = Mean squares; Effect size = partial η^2^

**p* < 0.05. ***p* < 0.01. ****p* < 0.001

To confirm that our stimuli were internally consistent and that Black compared to White participants’ responses did not differ markedly as a function of the race of the person in each clip, we compared emotion ratings in response to White and Black targets separately within each condition. Hence, we conducted four separate 2 (participant group: Black, White) x 2 (target race: Black, White) x 5 (Emotion: compassion, personal distress, moral indignation, guilt, shame) mixed factorial ANOVAs, one for each condition, using emotion change scores from the neutral condition. In these analyses, significant interactions between participant group and target race would suggest that White and Black participants’ responses differed within each condition as a function of the race of the target (i.e., victims and perpetrators).

In the victim forgiving (VF) condition, only the main effect of emotion was significant, *F*(4, 31) = 19.60, *p* < 0.001. Of significance, is that the two-way interaction between participant group and target race was nonsignificant (*p* = 0.78), as was the three-way interaction between participant group, target race, and emotion (*p* = 0.18). Participant responses therefore did not differ markedly as a function of target race.

In the victim unforgiving (VU) condition, the main effect of emotion was again significant, *F*(4, 31) = 11.58, *p* < 0.001, with no other main effects reaching significance. In addition, the two-way interaction between participant group and target race, as well as the three-way interaction between participant group, target race, and emotion were again nonsignificant (*p*s > 0.30). Hence, participant responses did not differ markedly as a function of target race for this condition.

In the perpetrator apologetic (PA) condition, the main effect of emotion was again significant, *F*(4, 31) = 22.06, *p* < 0.001, while no other main effects reached significance. As in the previous conditions, the interactions between participant group and target race, and between participant group, target race, and emotion were nonsignificant (*p*s > 0.19), suggesting no significant effects of target race.

Finally, in the perpetrator unapologetic (PU) condition, the main effect of emotion was significant, *F*(4, 31) = 14.05, *p* < 0.001. In addition, the main effect of target race was significant, *F*(1,34) = 5.62, *p* = 0.02, such that all participants’ emotion ratings were higher in response to White compared to Black perpetrators. Finally, the interaction between participant group and emotion reached significance, *F*(4,31) = 3.03, *p* = 0.03. This interaction could be explained by the fact that White participants’ ratings of guilt and shame in the PU condition were significantly greater than those of Black participants. No other interactions reached significance.

Taken together, these analyses confirm that Black and White participants’ emotion ratings did not differ significantly as a function of the race of victims and perpetrators portrayed in the video clips. The only significant effect for target race was observed in the PU condition, where Black and White participants’ emotion ratings were in the same direction (i.e., higher in response to White compared to Black perpetrators). We therefore felt confident that our participants’ emotion ratings within each condition were not affected in different ways by target race.

***Behavioral Data: Questionnaire Measures***

Table S4

*Zero-Order Correlations Between Participants’ Social Discrimination, Early Adversity, and Compassion Responses*

| Compassion | Everyday Discrim | CTQ Total | CTQ  E Abuse | CTQ  P Abuse | CTQ  S Abuse | CTQ  E Negl | CTQ  P Negl |
| --- | --- | --- | --- | --- | --- | --- | --- |
| VF | -.43**  [-.63, -.17] | -.43**  [-.65, -.15] | -.45**  [-.67, -.16] | -.32  [-.56, -.01] | -.20  [-.52, .21] | -.34*  [-.62, .01] | -.31  [-.55, .02] |
| VU | -.43**  [-.66, -.12] | -.26  [-.53, .03] | -.32  [-.57, -.04] | -.20  [-.45, .06] | -.13  [-.47, .29] | -.16  [-.49, .21] | -.13  [-.47, .26] |
| PA | -.46**  [-.70, .07] | -.38*  [-.66, -.08] | -.43**  [-.69, -.14] | -.35*  [-.64, -.05] | -.08  [-.46, .35] | -.32  [-.63, .03] | -.28  [-.60, .06] |
| PU | -.26  [-.54, .01] | -.02  [-.35, .30] | -.08  [-.43, .28] | -.11  [-.46, .24] | .15  [-.15, .46] | -.10  [-.45, .28] | -.04  [-.41, .32] |

*Note*. Data presented are correlation coefficients, with 95% bootstrap CIs in brackets.

CTQ = Childhood Trauma Questionnaire; CTQ Total = total scores; CTQ E Abuse = emotional abuse scores; CTQ P Abuse = physical abuse scores; CTQ S Abuse = sexual abuse scores; CTQ E Negl = emotional neglect scores; CTQ P Negl = physical neglect scores; Everyday Discrim = everyday discrimination scores; VF = victim forgiving; VU = victim unforgiving; PA = perpetrator apologetic; PU = perpetrator unapologetic.

**p* < 0.05. ***p* < 0.01.

***fMRI Data: Whole-Brain Contrasts***

Table S5

*Contrast Between Victim Forgiving and Neutral Condition (VF > Neu)*

|  |  | Coordinates | | | Cluster size^a^ |  |
| --- | --- | --- | --- | --- | --- | --- |
| Region | Hem. | *x* | *y* | *z* |  | *Max t* |
|  |  |  |  |  |  |  |
| Middle temporal gyrus and temporal pole | R | 45 | -25 | -2 | 6115 | 7.22 |
| Middle temporal gyrus & TPJ | L | -48 | -61 | 13 | 13292 | 5.86 |
| Temporal pole | L | -48 | 5 | -23 | 1593 | 4.92 |
| IFG | R | 51 | 20 | 13 | 1102 | 5.22 |
|  | L | -54 | 17 | 16 | 3634 | 7.76 |
| dmPFC | L/R | -3 | 44 | 37 | 838 | 4.36 |
| Precuneus | L/R | 0 | -58 | 28 | 1648 | 4.99 |
| Premotor cortex | L | -42 | -4 | 49 | 2143 | 5.46 |
| Occipital gyrus | R | 39 | -73 | -8 | 714 | 4.34 |
|  |  |  |  |  |  |  |

*Note.* Talaraich coordinates and *t*-score refer to the peak of each brain region. Reported clusters survived Monte Carlo cluster-level thresholding at an uncorrected *p* < 0.005 (min cluster size 567 mm^3^).

dmPFC = dorsomedial prefrontal cortex; IFG = inferior frontal gyrus; TPJ = temporoparietal junction

^a^Cluster size refers to the number of voxels (1x1x1 mm^3^ resolution of the iso-voxeled structural images).

Table S6

*Contrast Between Victim Unforgiving and Neutral Condition (VU > Neu)*

|  |  | Coordinates | | | Cluster size^a^ |  |
| --- | --- | --- | --- | --- | --- | --- |
| Region | Hem. | *x* | *y* | *z* |  | *Max t* |
|  |  |  |  |  |  |  |
| Middle temporal gyrus, temporal pole, and TPJ | R | 45 | -25 | -2 | 10589 | 7.22 |
| Middle temporal gyrus & TPJ | L | -48 | -61 | 13 | 12728 | 5.86 |
| Temporal pole | L | -48 | 5 | -23 | 934 | 4.92 |
| IFG | R | 51 | 20 | 13 | 2374 | 5.22 |
|  | L | -54 | 17 | 16 | 3734 | 7.76 |
| dmPFC | L/R | 3 | 56 | 28 | 1920 | 4.90 |
| Precuneus | L/R | 0 | -58 | 28 | 2594 | 4.99 |
| Premotor cortex | R | 42 | -4 | 49 | 673 | 3.80 |
|  | L | -42 | -4 | 49 | 3248 | 5.46 |
| Occipital gyrus | R | 45 | -64 | -5 | 2013 | 4.41 |
| Midbrain (tectum) | L/R | -3 | -28 | -2 | 591 | 4.41 |
|  |  |  |  |  |  |  |

*Note.* Talaraich coordinates and *t*-score refer to the peak of each brain region. Reported clusters survived Monte Carlo cluster-level thresholding at an uncorrected *p* < 0.005 (min cluster size 405 mm^3^).

dmPFC = dorsomedial prefrontal cortex; IFG = inferior frontal gyrus; TPJ = temporoparietal junction

^a^Cluster size refers to the number of voxels (1x1x1 mm^3^ resolution of the iso-voxeled structural images).

Table S7

*Contrast Between Perpetrator Apologetic and Neutral Condition (PA > Neu)*

|  |  | Coordinates | | | Cluster size^a^ |  |
| --- | --- | --- | --- | --- | --- | --- |
| Region | Hem. | *x* | *y* | *z* |  | *Max t* |
|  |  |  |  |  |  |  |
| Middle temporal gyrus | R | 45 | -31 | 4 | 650 | 4.16 |
| Temporal pole | R | 48 | 8 | -23 | 689 | 4.09 |
|  | L | -45 | 11 | -26 | 554 | 4.65 |
| IFG | L | -54 | 17 | 16 | 739 | 5.82 |
| Precuneus | R | 6 | -52 | 25 | 317 | 3.85 |
|  | L | -9 | -52 | 28 | 334 | 4.67 |
| Posterior parietal cortex | L | -42 | -58 | 19 | 2250 | 5.33 |
|  |  |  |  |  |  |  |

*Note.* Talaraich coordinates and *t*-score refer to the peak of each brain region. Reported clusters survived Monte Carlo cluster-level thresholding at an uncorrected *p* < 0.005 (min cluster size 216 mm^3^).

IFG = inferior frontal gyrus

^a^Cluster size refers to the number of voxels (1x1x1 mm^3^ resolution of the iso-voxeled structural images).

Table S8

*Contrast Between Perpetrator Unapologetic and Neutral Condition (PU > Neu)*

|  |  | Coordinates | | | Cluster size^a^ |  |
| --- | --- | --- | --- | --- | --- | --- |
| Region | Hem. | *x* | *y* | *z* |  | *Max t* |
|  |  |  |  |  |  |  |
| Middle temporal gyrus | R | 45 | -31 | 1 | 5928 | 7.43 |
| Middle temporal gyrus & TPJ | L | -51 | -37 | 4 | 17423 | 6.38 |
| TPJ | R | 61 | -46 | 19 | 894 | 4.50 |
| Temporal pole | R | 48 | 5 | -23 | 1597 | 5.44 |
|  | L | -45 | 11 | -27 | 1245 | 5.64 |
| IFG | R | 51 | 20 | 16 | 2403 | 5.27 |
|  | L | -51 | 20 | 13 | 7288 | 8.05 |
| dmPFC | L/R | -3 | 41 | 40 | 776 | 4.73 |
| Precuneus | L/R | 3 | -55 | 28 | 4292 | 6.64 |
| Premotor cortex | L | -42 | -4 | 43 | 2658 | 5.99 |
| Occipital gyrus | L | -45 | -70 | -2 | 649 | 4.43 |
| PAG | L | -3 | -25 | -2 | 1748 | 5.50 |
| Amygdala | L | -18 | -4 | -14 | 488 | 4.77 |
| Dorsal striatum and thalamus | R | 21 | -1 | 13 | 636 | 4.47 |
|  | L | -3 | -13 | 4 | 3003 | 6.15 |
|  |  |  |  |  |  |  |

*Note.* Talaraich coordinates and *t*-score refer to the peak of each brain region. Reported clusters survived Monte Carlo cluster-level thresholding at an uncorrected *p* < 0.005 (min cluster size 486 mm^3^).

dmPFC = dorsomedial prefrontal cortex; IFG = inferior frontal gyrus; PAG = periaqueductal gray; TPJ = temporoparietal junction

^a^Cluster size refers to the number of voxels (1x1x1 mm^3^ resolution of the iso-voxeled structural images).

Table S9

*Main Effect of Participant Group*

|  |  | Coordinates | | | Cluster size^a^ |  |
| --- | --- | --- | --- | --- | --- | --- |
| Region | Hem. | *x* | *y* | *z* |  | *Max t* |
|  |  |  |  |  |  |  |
| pSTS | R | 58 | -52 | 4 | 1013 | 4.74 |
| dACC | R | 9 | 32 | 25 | 931 | 4.58 |
| vmPFC | L/R | 6 | 44 | 1 | 1120 | 4.52 |
| dlPFC | L | -42 | 26 | 34 | 415 | 4.94 |
| Middle frontal gyrus | L | -27 | 47 | 13 | 784 | 4.18 |
|  | L | -24 | 35 | 22 | 414 | 4.54 |
| Premotor cortex | R | 23 | 2 | 52 | 556 | 4.43 |
| Precuneus | L/R | 0 | -37 | 34 | 358 | 4.10 |
| Inferior parietal lobe | L | -51 | -34 | 34 | 1188 | 4.82 |
| Thalamus/striatum | R | 18 | -11 | 11 | 739 | 4.74 |
|  |  |  |  |  |  |  |

*Note.* Talaraich coordinates and *t*-score refer to the peak of each brain region. Reported clusters survived Monte Carlo cluster-level thresholding at an uncorrected *p* < 0.005 (min cluster size 297 mm^3^).

dACC = dorsal anterior cingulate cortex; dlPFC = dorsolateral prefrontal cortex; pSTS = posterior superior temporal sulcus; vmPFC = ventromedial prefrontal cortex

^a^Cluster size refers to the number of voxels (1x1x1 mm^3^ resolution of the iso-voxeled structural images).

Table S10

*ANOVA Summary Table for Functionally Defined ROIs (Main Effect of Participant Group)*

| Source | *df* | MS | *F* | *p* | Effect size |
| --- | --- | --- | --- | --- | --- |
|  |  |  |  |  |  |
| pSTS |  |  |  |  |  |
| Condition (A) | 4 | 0.08 | 2.68 | 0.03* | 0.07 |
| Participant group (B) | 1 | 4.44 | 21.55 | < 0.001*** | 0.39 |
| A x B | 4 | 0.12 | 4.03 | 0.004** | 0.10 |
| vmPFC |  |  |  |  |  |
| Condition (A) | 2.82 | 0.02 | 0.47 | 0.69 | 0.01 |
| Participant group (B) | 1 | 0.64 | 28.32 | < 0.001*** | 0.45 |
| A x B | 2.82 | 0.04 | 0.13 | 0.29 | 0.04 |
| dACC |  |  |  |  |  |
| Condition (A) | 3.06 | 0.07 | 2.66 | 0.05 | 0.07 |
| Participant group (B) | 1 | 0.63 | 25.40 | < 0.001*** | 0.43 |
| A x B | 3.06 | 0.03 | 1.37 | 0.26 | 0.04 |
| Left dlPFC |  |  |  |  |  |
| Condition (A) | 4 | 0.07 | 3.73 | 0.006** | 0.10 |
| Participant group (B) | 1 | 0.61 | 24.17 | < 0.001*** | 0.42 |
| A x B | 4 | 0.03 | 1.51 | 0.20 | 0.04 |
|  |  |  |  |  |  |

*Note*. MS = Mean squares; Effect size = partial η^2^; dACC = dorsal anterior cingulate cortex; dlPFC = dorsolateral prefrontal cortex; pSTS = posterior superior temporal sulcus; vmPFC = ventromedial prefrontal cortex

**p* < 0.05, ***p* < 0.01. ****p* < 0.001

Table S11

*ANOVA Summary Table for Independent ROIs Analysis*

| Source | *df* | MS | *F* | *p* | Effect size |
| --- | --- | --- | --- | --- | --- |
|  |  |  |  |  |  |
| Left amygdala |  |  |  |  |  |
| Condition (A) | 2.99 | 0.20 | 7.10 | < 0.001*** | 0.17 |
| Participant group (B) | 1 | 0.11 | 3.26 | 0.08 | 0.09 |
| A x B | 2.99 | 0.01 | 0.26 | 0.86 | 0.01 |
| Right amygdala |  |  |  |  |  |
| Condition (A) | 4 | 0.13 | 4.03 | 0.004** | 0.11 |
| Participant group (B) | 1 | 0.00 | 0.05 | 0.83 | 0.00 |
| A x B | 4 | 0.02 | 0.46 | 0.77 | 0.01 |
| dmPFC |  |  |  |  |  |
| Condition (A) | 2.97 | 0.52 | 5.46 | <0 .001*** | 0.14 |
| Participant group (B) | 1 | 0.05 | 0.24 | 0.63 | 0.01 |
| A x B | 2.97 | 0.03 | 0.28 | 0.84 | 0.01 |
| Left TPJ |  |  |  |  |  |
| Condition (A) | 3.33 | 0.39 | 13.11 | < 0.001*** | 0.28 |
| Participant group (B) | 1 | 0.00 | 0.04 | 0.85 | 0.00 |
| A x B | 3.33 | 0.07 | 2.47 | 0.06 | 0.07 |
| Right TPJ |  |  |  |  |  |
| Condition (A) | 3.06 | 0.20 | 5.65 | < 0.001*** | 0.14 |
| Participant group (B) | 1 | 0.00 | 0.03 | 0.86 | 0.00 |
| A x B | 3.06 | 0.04 | 1.2 | 0.31 | 0.03 |
| Precuneus |  |  |  |  |  |
| Condition (A) | 3.17 | 0.24 | 7.75 | < 0.001*** | 0.19 |
| Participant group (B) | 1 | 0.05 | 1.17 | 0.29 | 0.03 |
| A x B | 3.17 | 0.02 | 0.54 | 0.67 | 0.02 |
| Left IFG |  |  |  |  |  |
| Condition (A) | 3.21 | 0.28 | 12.97 | < 0.001*** | 0.28 |
| Participant group (B) | 1 | 0.00 | 0.02 | 0.88 | 0.00 |
| A x B | 3.21 | 0.05 | 2.12 | 0.10 | 0.06 |
| Right IFG |  |  |  |  |  |
| Condition (A) | 3.05 | 0.21 | 8.36 | < 0.001*** | 0.20 |
| Participant group (B) | 1 | 0.09 | 1.77 | 0.19 | 0.05 |
| A x B | 3.05 | 0.03 | 1.35 | 0.26 | 0.04 |
|  |  |  |  |  |  |

*Note*. MS = Mean squares; Effect size = partial η^2^; dmPFC = dorsomedial prefrontal cortex; IFG = Inferior frontal gyrus; TPJ = temporoparietal junction

***p* < 0.01. ****p* < 0.001

***fMRI Data: Brain-Behavior Correlations***

Table S12

*Zero-Order Brain-Behavior Correlations*

|  |  | Black participants (*n* = 18) | | |  | White participants (*n* = 18) | | |
| --- | --- | --- | --- | --- | --- | --- | --- | --- |
| Region |  | Compassion | Everyday Discrim | CTQ  E Abuse |  | Compassion | Everyday Discrim | CTQ  E Abuse |
|  |  |  |  |  |  |  |  |  |
| L amygdala | VF  VU  PA  PU | -.46  -.22  .21  -.44 | .38  .37  -.05  .13 | .35  .21  .01  .20 |  | -.15  -.26  -.06  -.18 | .50*  .33  .58*  .36 | .16  .17  .17  .21 |
|  |  |  |  |  |  |  |  |  |
| R amygdala | VF  VU  PA  PU | -.12  -.03  -.08  -.47* | .16  .02  -.02  -.28 | .26  -.13  .00  -.10 |  | -.27  -.42  -.36  -.53* | .47*  .54*  .64**  .60* | .20  .28  .30  .34 |
|  |  |  |  |  |  |  |  |  |
| L TPJ | VF  VU  PA  PU | -.31  -.53*  -.44  -.29 | .48*  .67**  .34  .46 | .65**  .66**  .58*  .57* |  | -.23  .11  .01  .04 | .08  -.05  .23  .12 | .15  .10  .35  .18 |
|  |  |  |  |  |  |  |  |  |
| R TPJ | VF  VU  PA  PU | -.14  -.33  -.20  .05 | .21  .20  -.05  -.21 | .27  .24  .07  .01 |  | -.01  .30  .17  .46 | -.10  -.24  .07  -.01 | .13  -.08  .27  .01 |
|  |  |  |  |  |  |  |  |  |
| L IFG | VF  VU  PA  PU | -.30  -.50*  -.20  -.55* | .37  .64**  .32  .56* | .10  .19  .04  .26 |  | -.29  -.13  -.02  -.18 | -.04  -.19  .02  .08 | -.14  -.19  -.06  .01 |
|  |  |  |  |  |  |  |  |  |
| R IFG | VF  VU  PA  PU | -.01  -.39  .05  -.21 | .15  .26  .21  .41 | .04  .08  .06  .06 |  | -.02  -.04  -.04  -.19 | -.20  -.34  -.08  -.20 | -.12  -.19  .08  -.07 |
|  |  |  |  |  |  |  |  |  |

*Note*. Data presented are correlation coefficients.

CTQ E Abuse = emotional abuse scores; Everyday Discrim = everyday discrimination scores; IFG = inferior frontal gyrus; TPJ = temporoparietal junction; VF = victim forgiving; VU = victim unforgiving; PA = perpetrator apologetic; PU = perpetrator unapologetic

**p*_(2-tailed)_ < 0.05. ***p*_(2-tailed)_ < 0.01.

Table S13

*Simultaneous Regressions Predicting Brain Activation as a Function of Social Discrimination and Compassion for the Perpetrator Unapologetic (PU) Condition*

|  | Black participants | | |  | White participants | |
| --- | --- | --- | --- | --- | --- | --- |
|  | L IFG  (*R*^2^ = 0.55, *p* = 0.004) | | |  | L IFG  (*R*^2^ = 0.03, *p* = 0.79) | |
|  | *β* | | *p* |  | *β* | *p* |
|  |  |  |  |  |  |  |
| Everyday Discrimination | **0.53** | | **0.02** |  | 0.02 | 0.94 |
| Compassion | -0.38 | | 0.07 |  | -0.17 | 0.54 |
|  |  |  |  |  |  |  |

*Note*. IFG = inferior frontal gyrus

**References**

1 Lamm, C., Decety, J. & Singer, T. Meta-analytic evidence for common and distinct neural networks associated with directly experienced pain and empathy for pain. *Neuroimage* **54**, 2492-2502, doi:10.1016/j.neuroimage.2010.10.014 (2011).

2 Schurz, M., Radua, J., Aichhorn, M., Richlan, F. & Perner, J. Fractionating theory of mind: A meta-analysis of functional brain imaging studies. *Neurosci Biobehav Rev.* **42**, 9-34, doi:10.1016/j.neubiorev.2014.01.009 (2014).
